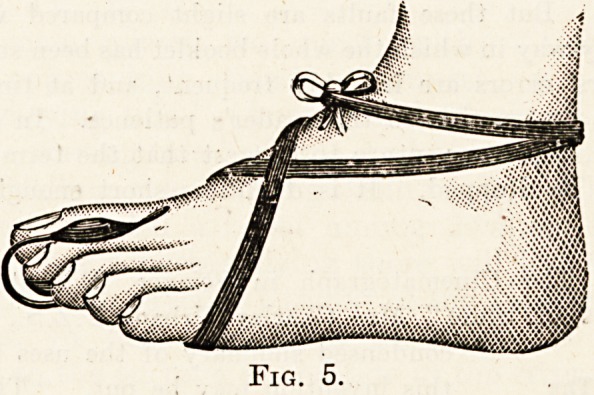# New Appliances and Things Medical

**Published:** 1907-09-14

**Authors:** 


### NEW APPLIANCES AND THINGS MEDICAL.

[We shall be glad to receive at our Office, 28 & 29 Southampton Street, Strand, London, W.C., from the manufacturers, specimens of all new preparations and appliances which may be brought out from time to time.]

### ORTHOPÆDIC APPLIANCES.

(K. R. Schramm and Co., 116 Albany Street, Regent's Park, N.W.)

This firm has submitted for experiment and report some of their orthopædic appliances, including their Valgus

Fig. 1.

Sock, designed to assist in supporting the arch of the foot when it is weakened by debility or injury. We have tried them in several cases, with markedly favourable results. One patient, a lady, slightly adipose and heavy on the feet, was obliged to stand in a shop for long hours together. The prolonged strain on the arch of the foot weakened it, and caused a condition amounting to absolute lameness. Schramm's Valgus Socks were ordered, with the result that the condition was rectified and the patient

Fig. 2.

was enabled to do her work in comfort. In another instance the socks were supplied to a patient who had both fibulæ fractured and the arch of the foot greatly broken down. When first seen after the fractures had healed and united, he walked upstairs with difficulty, and on going down he was obliged to crawl backwards, as he could not bend the foot. He was provided with Schramm's Valgus Socks, and the pain of walking was almost immediately removed. A few weeks later he could bend the arch and walk easily. We have tested their usefulness in other cases with equally striking results. The socks are ventilated, non-corrosive, springy, and durable, and can be worn inside the ordinary boot without discomfort.

This firm has also sent to us a bunion lever, an effective appliance for correcting certain deformities of the hallux and removing pressure and friction from bunions.

Fig. 3.

Among other orthopœdic appliances provided by this firm is a concealed spring which can be fitted to the inside of the boot, and thereby to serve as a support for weak ankles in ladies and children. It is jointed to correspond with the articulation of the ankle joint, and is devised to restore the equilibrium of the foot and the strength of the ankle.

All the work of this firm which has passed through our hands deserves the highest commendation for ingenuity, originality, and excellent workmanship.

One of the specially note-worthy appliances is an automatic arrangement for straightening bent toes and for relieving them from pressure and sores. The firm provides a hammertoe device which can be used for walking, and is

Fig. 4.

designed to counteract contraction and pressure during the day time. The appliance derives its straightening power from a flattened elastic band. It can be conveniently worn in the boots and is made in various sizes.

A different method is used while the patient is in bed. This contrivance has a double spring action, is carefully

Fig. 5.

padded, and can be comfortably attached to the toe by means of the spring action and to the foot by an arrangement of tapes. It can be used to correct deformities of any toe without discomfort. We have made several trials of Schramm's ingenious devices, and have not been disappointed by the results obtained.

### TERTIS'S "BORAMA" DRESSING.

(Messrs. Krohne & Sesemann, 37 Duke Street, Manchester Square, London, W.)

We have carefully tested a sample of this dressing submitted to us. It consists of porous boric amadou, backed with a thin layer of double cyanide gauze. A convenient-sized piece is cut off the sheet, according to the directions enclosed in each packet, and is then placed in boiling water. The now pliant dressing is cooled in boric lotion and applied over the wound, gauze being superimposed if desired. A dressing so applied over a scalp wound remained in position for twenty hours, and was then easily removed on wetting with lukewarm distilled water; and another, used over a small granulating wound, was equally satisfactory. "Borama" shows no tendency to adhere to the wound, and it should prove of particular service in the dressing of skin grafts.

## Figures and Tables

**Fig. 1. f1:**
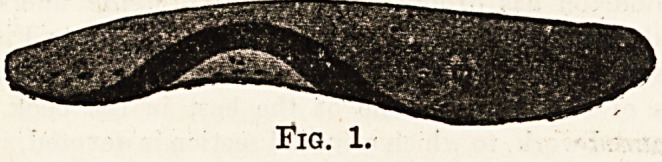


**Fig. 2. f2:**
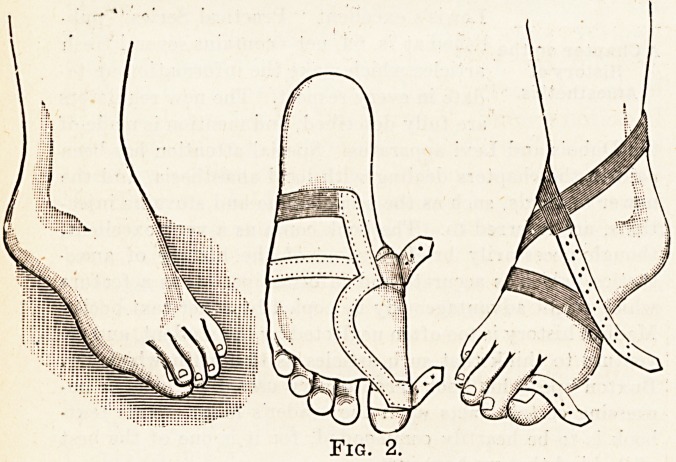


**Fig. 3. f3:**
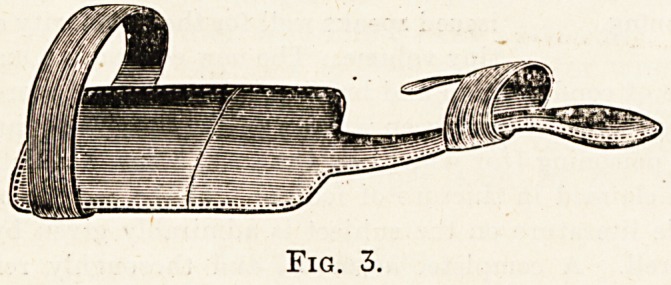


**Fig. 4. f4:**
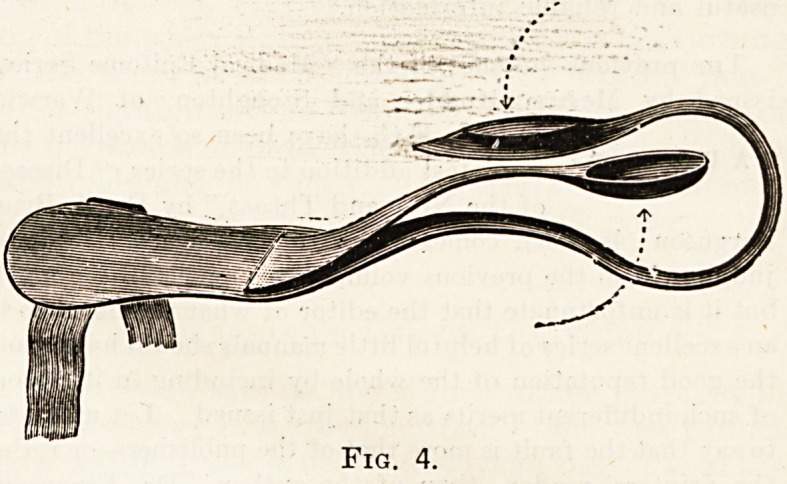


**Fig. 5. f5:**